# Looking inside the black box: a theory-based process evaluation alongside a randomised controlled trial of printed educational materials (the Ontario printed educational message, OPEM) to improve referral and prescribing practices in primary care in Ontario, Canada

**DOI:** 10.1186/1748-5908-2-38

**Published:** 2007-11-26

**Authors:** Jeremy M Grimshaw, Merrick Zwarenstein, Jacqueline M Tetroe, Gaston Godin, Ian D Graham, Louise Lemyre, Martin P Eccles, Marie Johnston, Jillian J Francis, Jan Hux, Keith O'Rourke, France Légaré, Justin Presseau

**Affiliations:** 1 Clinical Epidemiology Program, Ottawa Health Research Institute, Ottawa, Canada; 2 Institute of Population Health, University of Ottawa, Ottawa, Canada; 3 Institute of Clinical Evaluative Sciences, Toronto, Canada; 4 KT Program, University of Toronto, Canada; 5 School of Nursing, University of Laval, Quebec City, Canada; 6 School of Nursing, University of Ottawa, Ottawa, Canada; 7 School of Psychology, University of Ottawa, Ottawa, Canada; 8 Centre for Health Services Research, University of Newcastle upon Tyne, Newcastle upon Tyne, UK; 9 Department of Psychology, University of Aberdeen, Aberdeen, UK; 10 Health Services Research Unit, University of Aberdeen, UK; 11 Department of Family Medicine, University of Laval, Quebec City, Canada; 12 Canadian Institute of Health Research, Ottawa, Canada

## Abstract

**Background:**

Randomised controlled trials of implementation strategies tell us whether (or not) an intervention results in changes in professional behaviour but little about the causal mechanisms that produce any change. Theory-based process evaluations collect data on theoretical constructs alongside randomised trials to explore possible causal mechanisms and effect modifiers. This is similar to measuring intermediate endpoints in clinical trials to further understand the biological basis of any observed effects (for example, measuring lipid profiles alongside trials of lipid lowering drugs where the primary endpoint could be reduction in vascular related deaths).

This study protocol describes a theory-based process evaluation alongside the Ontario Printed Educational Message (OPEM) trial. We hypothesize that the OPEM interventions are most likely to operate through changes in physicians' behavioural intentions due to improved attitudes or subjective norms with little or no change in perceived behavioural control. We will test this hypothesis using a well-validated social cognition model, the theory of planned behaviour (TPB) that incorporates these constructs.

**Methods/design:**

We will develop theory-based surveys using standard methods based upon the TPB for the second and third replications, and survey a subsample of Ontario family physicians from each arm of the trial two months before and six months after the dissemination of the index edition of ***informed***, the evidence based newsletter used for the interventions. In the third replication, our study will converge with the "TRY-ME" protocol (a second study conducted alongside the OPEM trial), in which the content of educational messages was constructed using both standard methods and methods informed by psychological theory. We will modify Dillman's total design method to maximise response rates. Preliminary analyses will initially assess the internal reliability of the measures and use regression to explore the relationships between predictor and dependent variable (intention to advise diabetic patients to have annual retinopathy screening and to prescribe thiazide diuretics for first line treatment of uncomplicated hypertension). We will then compare groups using methods appropriate for comparing independent samples to determine whether there have been changes in the predicted constructs (attitudes, subjective norms, or intentions) across the study groups as hypothesised, and will assess the convergence between the process evaluation results and the main trial results.

**Trial registration number:**

Current controlled trial ISRCTN72772651

## Background

Recognition of the knowledge translation (KT) gap has led to increased interest in more active KT strategies. Over the past five years a considerable body of KT research has developed [1,2]. This research demonstrates that professional behaviour change interventions can be effective. However the effectiveness of interventions appears to vary across different clinical problems, contexts, and organizations, presumably due to the presence of different barriers and enablers to KT. Current quantitative evaluations of professional behaviour change strategies provide little insight into the causal mechanisms through which interventions lead to behaviour change and how they are moderated by different barriers and enablers to KT. This limits the ability to generalise from the findings of individual studies to other clinical problems, contexts and organisations. One of the challenges for KT researchers is to develop methods for exploring causal mechanisms alongside rigorous evaluations of different strategies.

## The Ontario Printed Educational Materials (OPEM) trial

The OPEM trial (PI – MZ, Co-investigators JG, JH) is a large factorial cluster randomised trial [3]. Participants will be randomised to one of four groups (control, short directive messages only, long discursive messages only, and both short and long messages). The messages will be embedded in the ***informed ***newsletter. This is produced by the Institute of Clinical Evaluative Sciences (ICES) (Ontario), and is a free, well regarded evidence-based practice synopsis, mailed quarterly since 1994 to 9,825 subscribers in Ontario, including all family practitioners (except 20 who opted to be removed from the mailing list). The short directive educational messages will be produced on a postcard-sized card stapled to the outside of ***informed***. The long educational messages will be produced as a two-page insert into ***informed ***(indistinguishable from the rest of the periodical in size, style and editing) excluding the directive statements and including more background, an evidence-based guideline, and references. OPEM will involve three replicated randomized trials in three successive editions of ***informed ***for three separate tracer conditions (assertive hypertension and cholesterol treatment in diabetic patients, regular diabetic retinopathy screening, and use of thiazide diuretics in the initial management of hypertension). Routinely collected administrative data (OHIP, ODB and CIHI data) available within ICES will be used to measure changes in professional behaviour for the four quarters before and after each intervention.

### Process evaluations alongside randomized trials of professional behavior change strategies

OPEM will be the largest and most rigorous evaluation of printed educational materials to date. It will tell us whether (or not) dissemination of printed educational materials results in changes in professional behaviour but nothing about the causal mechanisms that produce any change. This would not be an issue if we expected that the intervention would have a uniform effect across different conditions that could be generalised to practitioners outside of Ontario. However the current evidence base [4] indicates that the effects of interventions do appear to vary by condition, professional group, and context, presumably because the causal mechanisms of the interventions are modified in the presence of different barriers and enablers. Therefore, the interpretation of the results of the OPEM trial and assessment of its likely generalisability would be enhanced if we had additional information about the causal mechanisms through which the intervention worked, and how these were modified in the presence of different barriers and enablers. There is increasing recognition of the value of process evaluations alongside trials of complex interventions such as professional behaviour change interventions. Commonly, process evaluations have utilised qualitative methods to explore participants' attitudes toward and experiences of study interventions. For example, ME and JMG conducted a nested qualitative study alongside a randomised trial of computerised decision support for chronic disease management in UK primary care that identified that the intervention largely failed because of poor software implementation that was not integrated into family practitioners' work patterns [5,6]. Qualitative process evaluations provide valuable information about context-specific insights that can help interpret the results of an individual trial, but may be less helpful in predicting the likely generalisability of findings due to the lack of standardised constructs and measurements. In contrast, behavioural sciences have carefully developed and operationalised theories concerning determinants of behaviour and behaviour change. These standard definitions of constructs and measurement methods may be useful for exploring causal mechanisms of interventions and barriers and enablers to knowledge translation.

Theory-based process evaluations collect data on theoretical constructs alongside randomised trials to explore possible causal mechanisms and effect modifiers. This is akin to measuring intermediate endpoints in clinical trials to further understand the biological basis of any observed effects (for example, measuring lipid profiles alongside trials of lipid lowering drugs where the primary endpoint could be reduction in vascular related deaths). Ferlie and Shortell [7] have suggested four levels at which knowledge translation interventions might operate: the individual health professional; health care groups or teams; organisations providing health care; and the larger health care system or environment in which individual organizations are embedded. Different types of theory will be relevant to interventions at different levels. For example, psychological theories will be more relevant to interventions directed at individuals and teams, theories of organisational change will be more relevant to interventions directed at hospitals or trusts, and so on. A full scientific rationale for interventions to translate research findings into clinical practice requires exploration of theories relevant to each of these four levels.

## Aims and objectives

1. To conduct a theory-based process evaluation alongside the OPEM trial.

2. To advance the methodology of conducting theory-based process evaluations alongside randomised trials of professional behaviour change strategies

The specific objectives of the project are:

1. To develop theory-based survey instruments based upon the TPB (Phase I)

2. To conduct pre- and post-intervention postal surveys among family physicians in Ontario using the survey instruments for two of the OPEM study conditions (Phase II).

3. To analyse whether the OPEM interventions lead to significant improvements in theoretical constructs of the TPB (intentions, attitudes, subjective norms, perceived behavioural control) (Phase III).

4. To test the convergence of the results of the OPEM main trial and the theory-based process evaluation (Phase IV).

## Methods/design

### Project overview

As described earlier, OPEM was originally conceived as a two-by-two factorial design. This design was modified for the second and third iteration, transforming it into an incomplete two-by-three factorial randomised trial, for reasons documented in the OPEM trial protocol [3]. In the second iteration, the additional two groups had a reminder note added to the short directive message, formatted as a pad of patient-aimed reminder slips (short directive and pad, short directive and pad plus long discursive message). In the third iteration, the additional two groups had an outsert message developed based on the TPB, in comparison with the "standard" short messages similar to those developed for the first two iterations. The development of the psychologically informed outsert message is described in the "TRY-ME" Study Protocol [8]. Table 1 describes the groups in each iteration.

**Table 1:** Description of the intervention groups within the two replicates of the OPEM Trial

**REPLICATE 2: Retinal screening for patients with diabetes**			
		*Insert*	*No insert*

**OUTSERT**	**Patient Reminder Note**	1. Insert & Outsert & Patient Reminder	2. Outsert & Patient Reminder Note
	**No Patient Reminder Note**	3. Insert & Outsert	4. Outsert only
**NO OUTSERT**		5. Insert Only	6. No PEM

**REPLICATE 3: Diuretics for first-line treatment of hypertension**			

		*Insert*	*No insert*

**OUTSERT**	**Theory-based Outsert**	1. Insert & Theory-based Outsert	2. Theory-based Outsert Only
	**Non-theory-based outsert**	3. Insert & Non-theory-based Outsert	4. Non-theory-based Outsert only
**NO OUTSERT**		5. Insert Only	6. No PEM

Theory-based process evaluations collect data on theoretical constructs alongside randomized trials to explore potential causal mechanisms. We hypothesize that the OPEM intervention causes changes in physicians' intentions due to improved attitudes or subjective norms with little or no change in perceived behavioural control. We will test this hypothesis using the TPB model that incorporates these constructs [9]. We will develop theory-based surveys using standard methods [10,11] based upon the TPB for the second and third replications, and survey a subsample of recipients from each arm of the trial two months before and six months after the dissemination of the index edition of ***informed (***given the timing of the funding application and decision, we were unable to conduct a theory-based replication for the first replication of the OPEM trials). We will use Dillman's total design method to maximise response rates [12]. Analysis initially will assess the internal reliability of the measures, and use regression to explore the relationships between predictor and dependent variable (intention to undertake the recommended practice). We will then compare groups using methods appropriate for comparing independent samples (t-tests to compare two groups, analysis of covariance to compare groups adjusting for differences in baseline performance) to determine whether there have been changes in the predicted constructs (attitudes, subjective norms or intentions) across the study groups as hypothesised. We will use the Cox-Wermuth method (described below) for exploring dependencies and associations within systems to explore whether there is convergence between the theory-based process evaluation results and the main trial results.

#### Phase 1. Development of survey instruments

We will develop the survey instrument using standard methods [10]. TPB instruments can be developed based upon direct measures of the TPB constructs, or based on belief measures of the TPB constructs. The direct measures are relatively straightforward to develop and are relatively short and easy to complete (three to five items per construct, i.e., a total of 15–20 items). In contrast, belief-based measures are more complex to develop, and are considerably longer and more complex to complete. Belief-based measures are likely to be most beneficial if the aim is content-focused, that is if the goal is to identify specific beliefs that could be effectively targeted by an educational intervention. In the present study, the aim is to identify the causal mechanisms through which the OPEM interventions do or do not work; direct measure surveys are generally sufficient for this purpose and are more likely to be acceptable to physicians especially for repeated surveys.

We therefore plan to use a direct measure survey. Careful specification of the behavior is essential during the development of TPB surveys. We will decide on the specification of the behavior based on drafts of the short and long educational messages and the primary outcome for the OPEM trial. The *specified behavior *will be defined in terms of the TACT (target, action, context and time) principle (for example, prescribing diuretics as the first line treatment in newly diagnosed elderly hypertensive patients in the next six months). We will measure generalized intention via respondents' responses to three items measured on a seven point response format ("I will <behaviour>", " I plan to <behaviour>", and "I intend to <behaviour>". For example, "I plan to prescribe thiazide diuretics in newly diagnosed elderly hypertensive patients in the next six months"). Our direct measure of attitude will use a common stem (for example, "For me, prescribing thiazide diuretics in newly diagnosed elderly hypertensive patients in the next six months would be: ...") and four items using evaluative bipolar adjectives with a seven point response format (for example, "good practice...bad practice"). We will use both instrumental items (reflecting whether the behavior achieves something, for example, "<behaviour> is necessary..... unnecessary") and experiential items (reflecting how the respondents feel when performing the behaviour, for example, "satisfying..... not satisfying"). The specification of the bipolar adjectives will be considered carefully during both the development and pilot testing of the interview. Our direct measure of subjective norms will involve three items with a seven point response format anchored by "strongly agree" to "strongly disagree" (for example, "Most people who are important to me think that <behaviour>", "It is expected of me that I <behaviour>", and "I feel under social pressure to <behaviour>", for example, "I think most general practitioners/family physicians would approve of me prescribing thiazide diuretics in newly diagnosed elderly hypertensive patients in the next six months"). Our direct measure of perceived behavioral control will involve four items with a seven point response format. We will use items relating to both difficulty (whether the respondent thinks that she can actually do the behavior, e.g., "Doing the <behavior> is difficult for me","I am confident that I could <behavior>"), and controllability (whether the respondent believes that she is in control of the behavior, e.g., "There are factors outside of my control that would prevent me from prescribing thiazide diuretics in newly diagnosed elderly hypertensive patients in the next six months"). We will distribute items throughout the questionnaire so that questions used to assess different measures are interspersed to avoid a response set bias. We will also measure habit (past behaviour) by asking the respondents: "Thinking about your last ten elderly patients newly diagnosed with uncomplicated hypertension, for how many of them did you prescribe thiazide diuretics as a first-line drug treatment?" The survey will also include demographic questions to provide information about the sample.

We anticipate that each survey will have 15–20 items and could be completed by practitioners in 5 – 7.5 minutes. Initial drafts of each survey will be circulated within the OPEM, and OPEM theory-based process evaluation project teams to ensure face and content validity. We will pilot each survey with six family physicians using a semi-structured interview format.

### Scoring of measures

Measures of generalised intention, attitudes, subjective norms and perceived behavioural controls will be calculated as the mean of the measure item scores.

Copies of the survey instruments are available upon request.

#### Phase II. Postal survey implementation

The OPEM trial team will provide us with a sampling frame for the surveys. Physicians sampled for the first condition (regular diabetic retinopathy screening) will be excluded from the sampling frame for the second condition (diuretics for hypertension). The surveys will be administered using a modification of Dillman's tailored design method for mail surveys [12]. This will involve sending a cover letter with the initial survey mail to explain the purpose of the survey, why completing it is important, how the results might be used, and the confidentiality of survey results. A reminder post card will be sent at week two with a replacement questionnaire at weeks four and six. Respondents will be offered the option of faxing the survey back to us. Cummings et al found an average response rate of 61% in a random sample of studies using surveys mailed to physicians [13]. To help promote an acceptable response rate, the questionnaire will be kept to a maximum of two pages in length. In addition, we will provide $20 (CDN) to every physician who returns a completed questionnaire in recognition of the time required to complete the survey. Multiple studies have demonstrated that financial incentives increase response rates among both the public and physicians [11,13,14]. Physicians will be encouraged to return a blank questionnaire if they do not wish to participate in the study and will be deleted from the sampling frame. The pre-intervention surveys will be sent eight weeks before the distribution date for the relevant ***informed ***newsletter, and the post-intervention surveys will be sent to respondents of the pre-intervention survey six months after the distribution date.

Quality assurance procedures will be implemented to ensure the integrity of the survey data collection [15,16]. All aspects of the protocol will be elaborated in a detailed protocol manual for the study team. For the survey, a log record will be initiated and maintained to track the study status of participants throughout the mailings of the survey. They will be assigned a code number to be used on all subsequent study documentation to ensure confidentiality.

Data monitors to assess data entry accuracy will check a randomly selected sample (ten percent) of surveys. An error rate greater than 1% will be considered unacceptable, requiring all cases to be re-entered and rechecked.

#### Phase III. Planned analyses

We will test internal reliability of the measures using Cronbach's alpha. If internal consistency is <0.7, we will explore whether we can improve this by omitting any individual item. We will use regression to explore the relationships between predictor (attitudes, subjective norms, perceived behavioural control) and dependent variable (intention to undertake the recommended practice). If the dependent variable is markedly skewed, we will use generalized linear modelling regression to allow for this [17].

We will then compare groups using methods appropriate for comparing independent samples (t-tests to compare two groups, analysis of covariance to compare groups adjusting for differences in baseline performance) to determine whether there have been changes in the predictor constructs (attitudes, subjective norms, perceived behavioural control or intentions) across the study groups as hypothesised.

Further analysis will be informed and guided by the approach developed by D.R. Cox and N. Wermuth [18]. Their approach is directed more at the study of dependencies and associations with the objective of "understanding" the system under study, rather than just a "black box" empirical determination of the presence or absence of effects. This understanding is in the sense of gaining some knowledge of the underlying process, gaining some insight into the ability to predict in differing contexts, and relating the particular data under analysis to current knowledge of the field in question. The analyses proceeds by grouping variables into responses, intermediate responses, and explanatory variables, usually in blocks over time, and utilizing fairly standard and well-understood statistical regression methods to investigate the dependencies between blocks and within blocks. If the dependencies within blocks can be safely ignored, the approach is implemented with just a number of simple regression analyses, all involving univariate responses. For example, the regression methods can be a combination of linear and generalized linear regressions appropriate for the various responses, and non-linear if required to properly model the effects of various covariates. The approach offers an alternative to structural equations modelling that allows the use of standard statistical techniques and the interpretation of parameters as regression coefficients. Analysis will initially use multiple regression analysis to explore the relationships between predictor and dependent variable (intention to undertake the recommended practice). This analysis will allow us to explore whether there is convergence between the treatment effects of the theory-based process evaluation and the main trial results.

### Sample size considerations

A simple and often used approach to calculating the required sample size for two-by-two factorial trials is to calculate sample size for a two group study and then use the number per group for the four groups in the two-by-two factorial trial. In our case, using standard methods for continuous outcomes, we need 63 subjects per group to achieve 80% power of detecting an effect size of 0.5 standard deviations using a significance level of 5%, giving a total sample size of 252 for each experiment. Assuming a 50% response rate for each survey (pre- and post-intervention), we will mail the survey to 252 physicians per group to achieve this sample size (i.e. 50%, or 126 per group, complete the first survey and 50% of these, or 63 per group, complete the second survey).

We performed a simulation to further investigate and demonstrate the appropriateness of this simple sample size calculation for our study. In the simulation, we randomly generated scores for each of the four groups, equally for the null hypothesis and alternatively with the mean of the second and fourth groups 0.5 standard deviations larger (alternative hypothesis of one main effect for short directive messages and no interaction). This data was then analyzed as a two-by-two factorial experiment where significance first was determined for any effect (Global F test), and then if significant, significance for main effects was determined. We simulated these trials 10,000 times (to give a standard error less than 0.5%) and under the null hypothesis the Global F test was significant 4.97% of the simulations, while under the alternative hypotheses the Global F test was significant 92.45% of the simulations, and the test for main effects for short directive messages significant 99.80% of the simulations, to provide an observed power for the main effect of 92.27%. In an additional simulation in which the fourth group mean was set only 0.35 standard deviations larger (representing a negative interaction where 30% of the short directive message effect is negated by the addition of long discursive messages), the main effect for short directive messages was still significant 82.56% of the simulations. To take into account the change in the OPEM trial from four to six groups, the design was switched to a two-by-three design (outsert, insert, post-it note/theory-based outsert) that omitted observations of post-it/theory-based outsert without insert (six groups observed) and the survey was mailed to 252 physicians per group.

### Ethical Approval

This study has received approval from the Research Ethics Board at The Ottawa Hospital.

## Discussion

This is one of the first prospective theory-based process evaluations with both baseline and post-intervention measurement; it will contribute to both theoretical and methodological developments in implementation science. The process evaluation of the OPEM trial provided by our TPB-based surveys will permit an analysis of the causal mechanisms of any observed change in the two targeted behaviours. We anticipate that the results will be primarily of interest to KT researchers and behavioural scientists, and those in disciplines interested in the determinants of behaviour and behaviour change.

The major limitation of the study is our inability to link, at an individual health care professional level, the results of the theoretical measures and the clinical behaviours. This is similar to the meta-analysis context, where in a meta-regression the group average covariate score is regressed against observed group outcomes – but in our case the average covariate score is not based on the whole group, but just a subset of it that may be self-selected and somewhat non-representative [19,20]. This requires some explicit consideration of potential biases in any formal mediation analysis [21] The proposed Cox and Wermuth approach was developed in the context of potentially biased observational data with the intent of developing interpretations which aim to be explanatory in as deep a sense as is feasible.

We can envisage a number of different scenarios (Table 2):

**Table 2:** Possible scenarios about the congruence between the results of the OPEM trial and the theory-based process evaluation

		**Theory-based process evaluation result**	
		
		**+**	**-**
**OPEM trial results**	+	*A*	*B*
	-	*C*	*D*

### Scenario A: The OPEM trial observes improvements in clinical behaviours and the theory-based process evaluation observes improvements in our hypothesised mediators (attitudes, subjective norms and intentions)

This would suggest that the educational materials may have changed behaviour through our hypothesised mediators.

### Scenario B: The OPEM trial observes improvements in clinical behaviours and the theory-based process evaluation observes no improvements in our hypothesised mediators

There are four possible explanations: First. that the OPEM intervention operated through other mediating mechanisms. Second, that the theoretical measures that we used are not sensitive predictors of behaviour change (e.g., by resulting in data with limited variance). Third, that post intentional factors (not captured in our theoretical measures) mediated or moderated the effects of the intervention with the result that family practitioners acted even though they were not distinguished by any difference in intentions. Fourth, that there was a selection bias in the theory-based process evaluation with responders not being representative of the family practitioners in the OPEM trial.

### Scenario C: The OPEM trial observes no improvement in clinical behaviours and the theory-based process evaluation observes improvements in our hypothesised mediators

Again, there are three possible explanations. First, the intervention led to changes in the mediators that were not sufficient to result in behaviour change (a threshold hypothesis). Second, that post-intentional factors (not captured in our theoretical measures, for example, environmental or organisational barriers) mediated or moderated the effects of the intervention with the result that family practitioners did not (or could not) act upon their improved intentions (an intention-behaviour gap hypothesis). Third, there was a selection bias associated with responders being non representative of the family practitioners in the OPEM trial.

### Scenario D: The OPEM trial observes no improvement in clinical behaviours and the theory-based process evaluation observes no improvements in our hypothesis mediators

This would suggest that the intervention did not influence either our hypothesised mediators or the clinical behaviour (given available power to detect such, or the confidence interval ruling out important effects). If baseline measures of our hypothesised mediators are high, this might suggest that the barriers to evidence-based practice did not relate to knowledge, attitudes, and intentions, and therefore the intervention was unlikely to lead to improvements in clinical behaviour.

Information about potential barriers not relating to our hypothesised mediators will be captured in the open-ended questions of the surveys, and will allow us to explore whether family practitioners believe there are additional factors that might mediate or moderate the effects of the intervention. We will explore the extent of selection bias in responders to the theory-based process evaluation using routinely available data.

We anticipate that scenarios A and D are most likely, and plan to make predictions about the expected results of the OPEM trial based upon the theory-based process evaluation results that will be available before the OPEM trial results.

A further potential limitation of our study is that change in physician behaviour via printed educational materials is wholly dependent upon exposure to them. Dissemination of the inserts and outserts in ***informed ***does not guarantee that physicians will read them. However, in 1997 The Strategic Counsel Inc. contacted 500 Ontario physicians by phone to determine recall and readership of ***informed***. They found that 71% of respondents recalled receiving ***informed***, that 89% found it useful or very useful, and that 53% of those who recalled receiving it read most or every issue. This has important implications for this process evaluation, as potential changes on socio-cognitive constructs underlying behaviour are obviously dependent on exposure to and cognitive processing of the printed educational materials. This potential limitation is recognised in the pragmatic design of OPEM trial which is attempting to evaluate whether printed educational materials are likely to be effective in real world settings.

## Abbreviations

CIHI – Canadian Institute for Health Information

CIHR – Canadian Institutes of Health Research

ICES – Institute for Clinical Evaluative Sciences

ODB – Ontario Drug Benefit Program

OHIP – Ontario Health Insurance Plan

OPEM – Ontario Printed Educational Material

TPB – Theory of Planned Behaviour

## Competing interests

The author(s) declare that they have no competing interests.

## Authors' contributions

All authors contributed to the development of this study. All authors read and approved the final manuscript.

## References

[B1] Bero LA, Grilli R, Grimshaw JM, Harvey E, Oxman AD, Thomson MA (1998). Closing the gap between research and practice: an overview of systematic reviews of interventions to promote the implementation of research findings. The Cochrane Effective Practice and Organization of Care Review Group. BMJ.

[B2] NHS Centre for Reviews and Dissemination (1999). Getting evidence into practice. Effective Health Care Bulletin.

[B3] Zwarenstein M, Hux JE, Kelsall DL, Paterson JM, Grimshaw JM, Davis DA (2005). The Ontario Printed Educational Message Trial (OPEM). Implementation Science.

[B4] Grimshaw JM, Thomas RE, MacLennan G, Fraser C, Ramsay C, Vale L (2004). Effectiveness and efficiency of guideline dissemination and implementation strategies. Health Technol Assess.

[B5] Eccles M, Grimshaw J, Steen N, Parkin D, Purves I, McColl E (2000). The design and analysis of a randomized controlled trial to evaluate computerized decision support in primary care: the COGENT study. Fam Pract.

[B6] Rousseau N, McColl E, Newton J, Grimshaw J, Eccles M (2003). Practice based, longitudinal, qualitative interview study of computerised evidence based guidelines in primary care. BMJ.

[B7] Ferlie EB, Shortell SM (2001). Improving the quality of health care in the United Kingdom and the United States: a framework for change. The Milbank Quarterly.

[B8] Francis J, Grimshaw JM, Zwarenstein M, Eccles MP, Shiller S, Johston M, O'Rourke K, Presseau J, Tetroe J (2006). Testing a TheoRY-informed MEssage ("TRY-ME"): A Sub-trial Within a Randomized Controlled Trial of Printed Educational Materials (The Ontario Printed Educational Message (OPEM) Trial). Implementation Science.

[B9] Ajzen I (1991). The theory of planned behaviour. Organizational Behaviour and Human Decision Process.

[B10] Francis JJ, Eccles MP, Johnston M, Walker AE, Grimshaw JM, Foy R (2004). Constructing questionnaires based on the theory of planned behaviour. A manual for health services researchers.

[B11] Godin G, Kok G (1996). The theory of planned behavior: a review of its applications to health-related behaviors. Am J Health Promot.

[B12] Dillman D (2000). Mail and internet surveys. The tailored design method.

[B13] Cummings SM, Savitz LA, Konrad TR (2001). Reported response rates to mailed physician questionnaires. Health Services Research.

[B14] Field JL, Cadoret CA, Brown ML, Ford M, Greene SM, Hill J (2002). Surveying Physicians. Do components of the"Total Design Approach" to optimizing survey response rates apply to physicians?. Med Care.

[B15] Rabaneck L, Viscole CM, Horwitz RJ (1992). Problems in the conduct and analysis of randomized clinical trials. Archives of Internal Medicine.

[B16] Gilliss CL, Kulkin IL (1991). Monitoring nursing interventions and data collection in a randomized clinical trial. Western Journal of Nursing Research.

[B17] McCullagh P, Nelder JA (1999). Generalized linear models.

[B18] Cox DR, Wermuth N (1998). Multivariate dependencies: models, analysis, and interpretation.

[B19] Greenland S, O"Rourke K, Rothman KJ, Greenland S, Lash T (2007). Meta-Analysis. Modern Epidemiology.

[B20] Thompson SG, Higgins JP (2002). How should meta-regression analyses be undertaken and interpreted?. Stat Med.

[B21] Shadish WR, Cook TD, Campbell DT (2002). Experimental and Quasi-Experimental Designs for Generalized Causal Inference.

